# The paraflagellar rod of kinetoplastid parasites: From structure to components and function

**DOI:** 10.1016/j.ijpara.2009.10.005

**Published:** 2010-02

**Authors:** Neil Portman, Keith Gull

**Affiliations:** Sir William Dunn School of Pathology, University of Oxford, Oxford OX1 3RE, UK

**Keywords:** Flagella, Cilia, Paraflagellar rod, Trypanosomiasis, Kinetoplastida, Axoneme

## Abstract

The role of the eukaryotic flagellum in cell motility is well established but its importance in many other aspects of cell biology, from cell signalling to developmental regulation, is becoming increasingly apparent. In addition to this diversity of function the core structure of the flagellum, which has been inherited from the earliest ancestor of all eukaryotes, is embellished with a range of extra-axonemal structures in many organisms. One of the best studied of these structures is the paraflagellar rod of kinetoplastid protozoa in which the morphological characteristics have been well defined and some of the major protein constituents have been identified. Here we discuss recent advances in the identification of further molecular components of the paraflagellar rod, how these impact on our understanding of its function and regulation and the implications for therapeutic intervention in a number of devastating human pathologies.

## Graphical abstract

**Figure:**
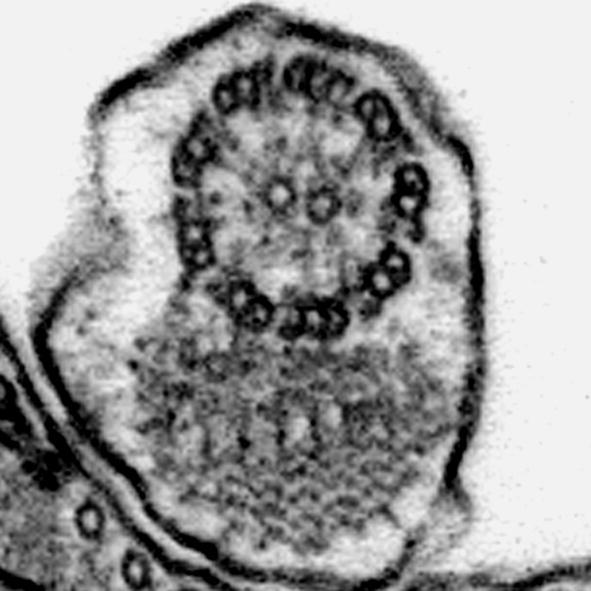

## 1 Introduction

**Fig. 1:**
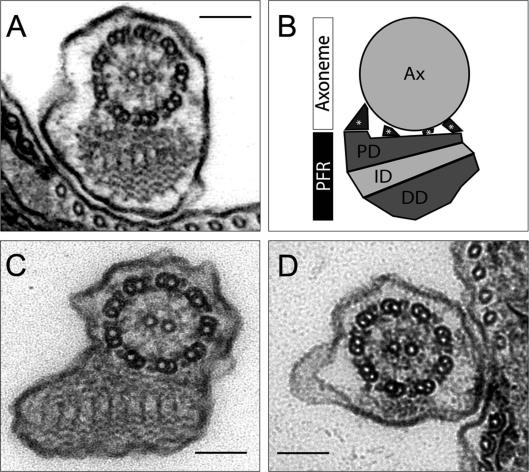
Transmission electron micrograph of transverse sections through (A) the *Trypanosoma brucei* flagellum and (B) the *Leishmania mexicana* flagellum. (C) A schematic diagram of the *T. brucei* flagellum showing the axoneme and the domains of the paraflagellar rod (PFR). (D) Transmission electron micrograph of a transverse section through the *T. brucei* flagellum 48 h after the induction of RNA interference against PFR2 showing the loss of a large portion of the PFR structure (*snl2* cell line). PD – proximal domain; ID – intermediate domain; DD – distal domain; Ax – axoneme; * – linking fibre bar = 100 nm. Parts of this figure are courtesy of Eva Gluenz and Amy Smith (B) and Sylvain Lacomble (D).

The paraflagellar rod (PFR) is one of several unique attributes characterising the biology of kinetoplastid protozoa and has served as a focus for speculation since its first identification by Keith Vickerman in 1962 (Vickerman, 1962). The PFR runs alongside the canonical 9 + 2 microtubular axoneme of Kinetoplastida and Euglenida (members of the monophyletic group, Euglenozoa), although in this review we will largely focus on studies involving the kinetoplastid family, Trypanosomatidae. Several early studies defined the ultrastructure of the PFR in various trypanosomatids (reviewed by Bastin et al., 1996b; Maga and LeBowitz, 1999) including species of *Trypanosoma*, *Phytomonas*, *Leishmania* and *Herpetomonas* (de Souza and Souto-Padron, 1980). Although the defining components of the PFR appear conserved throughout Kinetoplastida and Euglenida, the PFR ultrastructure is variable in size between species and in some cases a significantly reduced PFR is present. The Kinetoplastida PFR is a complex, trilaminar lattice-like structure with proximal, intermediate and distal domains defined (Fig. 1A–C). Transmission electron microscopy (TEM) reveals the proximal domain as a simple structure whilst the intermediate and distal domains show precise orientations of thick and thin filaments whose arrangement is often characteristic of the species (de Souza and Souto-Padron, 1980; Farina et al., 1986; Sant’Anna et al., 2005). The proximal domain of the PFR is linked to the axonemal microtubule doublets 4–7 by electron dense filaments (Farina et al., 1986) that are highly resistant to detergent and salt treatment but do yield to mild treatment with trypsin (Russell et al., 1983). In trypomastigote forms, and to some extent in epimastigote forms, the flagellum is attached along the cell body. In such cases, the proximal domain of the PFR is linked via filaments to the inner face of the flagellar membrane and then to the Flagellum Attachment Zone (FAZ). The PFR and axoneme maintain a precise orientation in regard to each other with the central pair microtubules having a consistent position (Gadelha et al., 2006). Mis-orientations or complete absence of the central pair can result from mutations in flagellar and basal body proteins (McKean et al., 2003; Branche et al., 2006; Gadelha et al., 2006; Ralston et al., 2006; Dawe et al., 2007) and there is some indication that variations can occur in overall position of the flagellum in relation to the cell body (Branche et al., 2006). However, evidence is still lacking as to whether there are specific changes to PFR structure or flagellar/cell body orientation in relation to flagellar wave progression.

## 2 Protein components of the PFR

A steadily increasing cohort of proteins is implicated as components of the PFR (Table 1). Early studies identified two highly abundant proteins, PFR1 and PFR2, which are now considered as the classic defining components of the PFR. Since then more than 40 additional proteins have been associated with the PFR through biochemical, bioinformatic and immunological techniques. The nature of these components provides increasing evidence for a PFR role in metabolic, regulatory and signalling functions. PFR components fall broadly into four groups: those involved in forming the lattice structure; those involved in adenine nucleotide signalling and metabolism; those involved in calcium signalling; and those for which a function has not yet been proposed.

### 2.1 The core components, PFR1 and PFR2

Comparison between flagella purified from the euglenid *Euglena gracilis* (possessing a PFR) and flagella from *Chlamydomonas reinhardtii* (no PFR), a flagellate green alga used for many years as a model for studying flagellar biology, identified two gel bands as likely highly abundant PFR components on the basis of their absence in the latter (Hyams, 1982). These proteins of 80 kDa and 69 kDa apparent molecular weight were named PFR1 and PFR2, respectively, and proof of their association with the PFR was provided by biochemical dissection studies in *Crithidia fasciculata* where flagellar preparations enriched for PFR components were shown to consist predominantly of two proteins of 76 kDa and 68 kDa after separation by SDS–PAGE (Russell et al., 1983). PFR1 and PFR2 were also subsequently observed by SDS–PAGE separation of purified flagella of *Herpetomonas megaseliae* (Cunha et al., 1984). The first molecular characterisation of these components was made in *Trypanosoma brucei* and suggested that these bands were encoded by one gene and the diversity resulted from differential protein folding (Schlaeppi et al., 1989). Subsequently, molecular evidence was presented for a simpler multigene explanation (Birkett et al., 1992) and a second gene was then characterised (Deflorin et al., 1994). The genome sequencing projects for *T. brucei*, *Trypanosoma cruzi* and *Leishmania major* now show that the genes encoding PFR1 and PFR2 are distinct but related and are present in separate tandem arrays (Berriman et al., 2005; El-Sayed et al., 2005; Ivens et al., 2005). Sequence analysis showed that both proteins are the result of a single gene duplication event that predates the divergence of Euglenida and Kinetoplastida and that all known extant instances of these proteins fit into a simplified and consolidated PFR1 and PFR2 nomenclature (Talke and Preisfeld, 2002; Gadelha et al., 2004). As a final confirmation, monoclonal antibodies and epitope tagging linked both proteins to a specific localisation in the PFR (Gallo and Schrevel, 1985; Bastin et al., 1996a; Kohl et al., 1999; Maga et al., 1999).

The PFR is a feature of all life-cycle stages of all Kinetoplastida studied to date with the exception of amastigote stages where the reduced flagellum does not emerge from the flagellar pocket. For many years it was thought that the endosymbiont-bearing Kinetoplastida placed in the *Crithidia*, *Blastocrithidia* and *Herpetomonas* genera did not adhere to this rule and did not possess a PFR (Freymuller and Camargo, 1981). However, recent work has shown that *Crithidia deanei* encodes and expresses a divergent PFR1 that is able to partially rescue a *Leishmania mexicana* PFR1 null mutant (Gadelha et al., 2005). Immunofluorescence using an antibody that recognises PFR1 and PFR2 in various Kinetoplastida labelled the proximal portion of the *C. deanei* flagellum. Such endosymbiont-containing kinetoplastids had previously been characterised as lacking a PFR. Furthermore, careful examination of *C. deanei* flagella by TEM revealed the presence of a structure resembling a reduced proximal domain of the canonical tri-laminar PFR of other Kinetoplastida with a location consistent with the immunofluorescence labelling. Thus the PFR, in various guises, appears to be a consistent feature of all Kinetoplastida.

### 2.2 Other protein components

Early studies using monoclonal reagents clearly indicated the presence of components in addition to PFR1 and PFR2. The ROD1 monoclonal antibody, produced by injecting mice with detergent extracted *T. brucei* procyclic form cells as part of an extensive screen for cytoskeletal components (Woods et al., 1989), labelled the distal domain of the PFR by immunogold labelling and recognised two bands of 180 kDa and 200 kDa apparent molecular weight by Western blot. Cell fractionation, detergent extraction and proteolysis techniques similar to those used to demonstrate the association of PFR1 and PFR2 with the PFR in *C. fasciculata* (Russell et al., 1983) were also used to identify a set of putative components between 122 kDa and 147 kDa from the flagellum of *H. megaseliae* (Moreira-Leite et al., 1999). Early attempts to identify less abundant PFR components used antisera to known mammalian cytoskeletal components and often found cross-reactivity with the PFR (Schneider et al., 1988). Unfortunately, it became clear that the PFR is a highly antigenic structure and often showed reactivity to many pre-immune sera or cross-reactivity with specific polyclonal antibodies even when affinity purified. Although some studies showed similar banding patterns on Western blots, these were not translated into confirmed identities at the sequence level. The high cross-reactivity of the PFR with many antisera impedes experimental interrogation and great stringency in experimental design is required when using such reagents for localisation studies. In trypanosomes, however, some approaches have been able to use complex (polyclonal–polyspecific) antisera and reveal the molecular identity of PFR antigens. Complex antisera against detergent extracted *T. brucei* were used to screen a lambda-phage expression library of *T. brucei* cDNA clones (Woodward et al., 1994). One clone (5.20) was identified, antisera to which had a very similar labelling pattern to ROD1 by both immunogold TEM and Western blot analysis. Whether this cDNA encodes the antigen for ROD1 is still unknown but the repetitive 5.20 protein is now displayed in the genome project of *T. brucei* as Tb11.01.6740 and has been identified as part of a PFR2-dependent cohort of flagellar proteins (discussed below) (Portman et al., 2009).

A screen of both *T. brucei* whole cell extracts and cytoskeleton preparations with *T. brucei* infected bovine serum identified two candidate PFR proteins (Imboden et al., 1995). A serum fraction affinity purified against *T. brucei* proteins of >180 kDa strongly labelled the flagellum and screening of a cDNA expression library identified two high molecular weight repeat proteins named I2 and I17. By immunogold TEM an antibody raised against I2 labelled the whole of the PFR while one raised against I17 specifically labelled the area between the PFR and the axoneme where the linking filaments are located.

The number of identified PFR proteins was increased by a series of antibody and phage expression library screens to identify the proteins PAR3, PAR2, PAR1 and PAR4 in *T. cruzi* (Saborio et al., 1989; Fouts et al., 1998). PAR3 and PAR2 correspond to the known PFR components PFR1 and PFR2, respectively. Antibodies specific to PAR1 and PAR4 both label the flagellum of *T. cruzi* trypomastigotes. Bioinformatic analysis suggested the presence of open reading frames potentially encoding homologues of these proteins in the genomes of *T. brucei* and *L. mexicana* and that PAR1 may be a divergent member of the PFR1/2 family. A proteomic analysis of the *T. brucei* flagellum (Broadhead et al., 2006) confirmed the expression and flagellar localisation of these proteins in this organism and more recently we have shown that in *T. brucei* PAR1 has a localisation consistent with the PFR and is not assembled into the flagellum after RNA interference (RNAi – a mechanism that inhibits gene expression) ablation of PFR2 (discussed below) (Portman et al., 2009).

Analysis of the Tritryp genomes (*T. brucei*, *T. cruzi* and *L. major*) (Berriman et al., 2005; El-Sayed et al., 2005; Ivens et al., 2005) allowed identification of PFR1/2 related sequences and production of Pfam domains. This process drew attention to two proteins that contain domains in common with PFR1 and PFR2 (Clark et al., 2005). These have been named PFR5 and PFR6 and have predicted molecular weights of 87 kDa and 75 kDa, respectively. PFR5 has an additional Src Homology 3 domain that is not present in other members of the PFR protein family. This domain binds proline-rich motifs and is involved in the formation of protein complexes although whether PFR5 has a role in the organisation of the PFR structure remains to be determined. The authors showed that transcripts for both PFR5 and PFR6 are significantly more abundant in *T. cruzi* trypomastigote and epimastigote life-cycle stages than in the amastigote stage, although in all stages the abundance of transcript was significantly lower than that observed for PFR1 or PFR2. As with PAR1, PFR5 was identified in a proteomic analysis of the *T. brucei* flagellum (Broadhead et al., 2006) and has been shown to be part of a cohort of proteins dependent on PFR2 for incorporation into the PFR (Portman et al., 2009).

All of these proteins apart from I17, which appears to be absent from the Leishmanial lineage, are represented by predicted open reading frames in the genome projects for *T. brucei*, *T. cruzi* and *L. major* (Berriman et al., 2005; El-Sayed et al., 2005; Ivens et al., 2005) reflecting a likely core composition for the PFR.

Intriguingly gamma tubulin was added to the growing list of potential PFR proteins with localisation to the flagellum of *Leishmania tropica* shown with monoclonal antibodies raised to peptides (Libusova et al., 2004). Gamma tubulin is normally associated with microtubule organising centres where it is involved in the nucleation of cytoplasmic and mitotic microtubules (reviewed by Raynaud-Messina and Merdes, 2007). It also localises to the basal body of the flagellum in *T. brucei* where it plays an essential role in the nucleation of the central pair microtubules (McKean et al., 2003). The possible role for gamma tubulin in the PFR remains cryptic. In the absence of functional data for a role for gamma tubulin in the PFR, this study exemplifies the difficulties encountered interpreting specificity of localisations when using even excellent monoclonal reagents. Our examination of the peptides used to raise the monoclonal antibodies in the context of recent *Leishmania* genome information (Ivens et al., 2005) reveals some level of possible cross-reactivity with other proteins.

Finally, recent work has shown that a novel myosin isoform, myosin XXI, that is restricted to kinetoplastids and down-regulated in the amastigote life-cycle stage localises to the flagellum of *Leishmania donovani* (Katta et al., 2009). Immunofluorescence analysis using a polyclonal antibody raised against recombinant MYO21 protein revealed a very strong signal at the proximal end of the flagellum with punctate staining throughout the cell body and along the length of the flagellum. Immunofluorescence double labelling with axonemal and PFR markers showed that the strong MYO21 signal at the base of the flagellum partially colocalised with the PFR suggesting a localisation in the extreme proximal domain of the PFR or in the area between the axoneme and the PFR. This proximal PFR localisation was also observed by immunogold thin section TEM and immunofluorescence analyses of GFP-tagged MYO21 domains showed that the tail region is necessary and sufficient for the observed localisation. As with many other identified PFR components, a function for MYO21 has yet to be determined although recently a role for actin dynamics in the formation of the kinetoplastid flagellum, and in particular the PFR, has been proposed (Tammana et al., 2008) and is discussed later.

### 2.3 Adenine homeostasis

Adenine nucleotides are involved or implicated in many aspects of flagellar function in diverse organisms. The major motor proteins of the flagellum, the outer dynein arm axonemal dyneins, contain AAA-ATPase domains and conformational changes to these proteins induced by the hydrolysis of ATP drive the microtubule sliding that generates the flagellar beat. Inner dynein arms are stimulated by low levels of ADP, suggesting that adenine nucleotide homeostasis is an important part of flagellar function (Yagi, 2000). Evidence from studies in many organisms suggests a pivotal role for the second messenger cAMP in the motility of spermatozoa (Kinukawa et al., 2006). It is thought that cAMP signalling is modulated by protein kinase A or via the Epac/Rap pathway and is proposed to be involved in the conversion of microtubule sliding to flagellar beat (Kinukawa et al., 2006 and references therein). There is also a body of work that supports a role for cAMP in the life cycle progression of various Kinetoplastida whereby changes in levels of intracellular cAMP induce differentiation (reviewed by Fenn and Matthews, 2007).*snl2* (Bastin et al., 2000) is an inducible mutant cell line of *T. brucei* with RNAi targeted against PFR2. Ablation of PFR2 leads to a failure of the cell to fully assemble a PFR. We hypothesised that minor components of the PFR could be identified by comparing flagella extracted from wild-type (PFR+) and *snl2*-induced (PFR−) cells due to these components entering the soluble phase in the absence of the PFR structure (Pullen et al., 2004). By comparing samples after separation by two-dimensional electrophoresis we identified a number of spots that were reduced after RNAi ablation of PFR2. Proteins from two of the spots were identified by tandem mass spectrometry as adenylate kinases (AKs) which were given the names TbADKA and TbADKB. Adenylate kinases are important enzymes in adenine nucleotide homeostasis and function to maintain the balance between the mono-, di- and tri-phosphate states. An epitope tagged copy of TbADKA was shown to localise along the whole length of the PFR. Both purified flagella and detergent extracted cytoskeletons were shown to have AK activity which was lost in preparations of *snl2* after RNAi induction, suggesting that AK activity in the insoluble fractions of the cell is restricted to the flagellum and in particular to the PFR. Importantly, although the residues involved in the structure and function of adenylate kinases in other organisms are conserved, both of these PFR adenylate kinases have 55 amino acid N-terminal extensions that show some sequence conservation between them. Peptides derived from these extensions were detected by tandem mass spectrometry showing that these are bona fide parts of the proteins and are not cleaved prior to incorporation into the PFR. In support of this, a truncation mutant of TbADKA lacking the N terminus failed to localise to the PFR and remained in the cytoplasm. This N-terminal sequence also proved to be sufficient for targeting a GFP fusion protein to the flagellum. This localisation was sensitive to non-ionic detergent treatment suggesting that other factors are involved in the stable incorporation of these proteins into the PFR structure. No effect on the morphology or function of the flagellum was detected after RNAi against both proteins simultaneously, although AK activity was reduced by 80% in cytoskeletons. This may be a result of residual AK activity being sufficient for normal flagellar function or that the specific circumstances under which these function are not preserved in culture conditions. Bioinformatic analysis of the N-terminal sequences identified homologous sequences in both human and *Caenorhabditis elegans* genome projects. Crucially, in these organisms the positive matches were also associated with genes encoding putative adenylate kinases. Whether this N-terminal sequence functions as a flagellar targeting signal in other organisms has yet to be determined, but the presence of a homologue of these unusual adenylate kinases in *C. elegans* may hint at a role in regulatory or signalling pathways, as all cilia produced by *C. elegans* are immotile. This proposed role may explain the lack of an observable phenotype in *T. brucei* when ADKs are ablated under culture conditions.

GRESAG4 adenylyl cyclase and the related bloodstream form calcium–regulated adenylyl cyclase ESAG4 have been shown to localise to the flagellar membrane of *T. brucei* (Paindavoine et al., 1992). These enzymes have divergent extracellular N-termini, a transmembrane domain and an intracellular catalytic domain that, due to the location of the enzyme, may interact with components of the axoneme and/or PFR. An additional study (D’Angelo et al., 2002) demonstrated the existence of a calcium-regulated adenylyl cyclase termed TczAC that is expressed in all life-cycle stages of *T. cruzi*. This enzyme exhibits dose-dependent stimulation by Ca^2+^ that is seemingly independent of calmodulin regulation despite lacking any identifiable calcium interacting domains. The authors showed that TczAC can dimerise via the catalytic domain and, interestingly, that it also interacts with either PFR1 or PFR2 by the same domain in Yeast-2-Hybrid analysis.

Further evidence for the role of cAMP in the Kinetoplastida flagellum, and particularly the PFR, came with the localisation of two cyclic nucleotide-specific phosphodiesterases to the PFR of *T. brucei* (Oberholzer et al., 2007b). These proteins, PDEB1 and PDEB2, show high similarity to each other at the catalytic domain but are divergent at their N-termini. Interestingly, this coincides with a differential localisation of the two proteins with PDEB1 being restricted to the PFR and PDEB2 localising mainly to the cytoplasm with a small portion present in the PFR. The PFR localisation of epitope tagged versions of both proteins was determined by immunofluorescence and immunogold TEM. This localisation was resistant to treatment with non-ionic detergent. This is in agreement with the presence of both proteins in a *T. brucei* flagellum proteome (Broadhead et al., 2006) generated from extracted flagella and suggests a tight association with the PFR. It has been shown that RNAi ablation of PFR2 results in an accumulation of un-polymerised PFR proteins at the tip of the new flagellum called the ‘blob’ (Bastin et al., 1999b). After ablation of PFR2, epitope tagged versions of both PDEB1 and PDEB2 were seen to localise to the blob (Oberholzer et al., 2007b). Importantly it was shown that ablation of both proteins by RNAi was lethal in the bloodstream form both in vitro (in culture) and in vivo (after infection into the mammalian host) although there was little effect on procyclic forms. This hypersensitivity of the bloodstream form is reminiscent of phenotypes resulting from the knockdown of other flagellar proteins in *T. brucei* (Broadhead et al., 2006; Ralston and Hill, 2006; Griffiths et al., 2007).

### 2.4 Calcium sensing

The role of Ca^2+^ signalling and calcium binding proteins in flagellar function has been well established over many years (Tamm, 1994). One of the most widely known calcium binding proteins, calmodulin, while having no intrinsic enzymatic activity, acts to transduce calcium signals to other proteins via calmodulin binding domains. Calmodulin has been shown to function in many parts of the axoneme including the radial spokes (Dymek and Smith, 2007) and central pair projections (Wargo et al., 2005). Other predicted calcium and calmodulin binding proteins have been identified in the radial spoke (Patel-King et al., 2004) and the outer dynein arm docking complex (Casey et al., 2003).

A non-calmodulin calcium binding protein was identified as being a major antigen recognised by Chagasic serum (serum from individuals infected with *T. cruzi*) in several studies. This 24 kDa protein (FCaBP) was purified and localised to the *T. cruzi* flagellum (Engman et al., 1989). Further work in *T. brucei* (Wu et al., 1992, 1994) identified a family of related flagellar non-calmodulin EF-hand calcium binding proteins that were named calflagins. An antibody that recognises the calflagins was shown to decorate the blob that accumulates at the tip of the new flagellum as a result of RNAi ablation of PFR2 in *T. brucei* (Bastin et al., 1999b). This finding may implicate at least one of the calflagins as a component of the PFR. Both FCaBP and the calflagins are dually acylated with myristoylate and palmitoylate and this was shown to be essential for localisation of FCaBP to the flagellar membrane (Godsel and Engman, 1999). Recent work has gone on to show that myristoylation alone targets calflagin Tb44 to the pellicular membrane and that palmitoylation, specifically by the palmitoyl acyl transferase TbPAT7, is then necessary for localisation to the flagellar membrane (Emmer et al., 2009). Furthermore, work on calflagin Tb24 showed that this protein displays biochemical characteristics of lipid-raft association (Tyler et al., 2009). Lipid rafts are areas of membrane that have increased liquid order and are associated with increased concentrations of sterols and sphingolipids (Brown and London, 2000). Tyler et al. (2009) provided evidence for increased liquid order in the *T. brucei* flagellar membrane and showed that in conditions under which lipid raft stability could be expected to be disrupted, flagellar localisation of calflagin Tb24 was also disrupted in both bloodstream form and procyclic form trypanosomes. As part of this study these authors also demonstrated an increased intensity of calflagin Tb24 staining in bloodstream forms at the anterior tip of the new flagellum where it contacts the old flagellum. This localisation at the tip of the new flagellum is consistent with the flagella connector of procyclic forms (Moreira-Leite et al., 2001) and with the predominant site at which new components are incorporated into the flagellar structure (Bastin et al., 2000). It is not currently clear whether calflagins are transported to the growing tip of the flagellum in an intraflagellar transport (IFT)-dependent manner although data have been presented suggestive of a role for lipid rafts in IFT (Tyler et al., 2009). The membrane association of FCaBP has been shown to be dependant on the calcium binding state of the protein (Godsel and Engman, 1999). In an environment in which calcium has been chelated, FCaBP dissociates from the membrane and enters the cytosolic fraction. This finding may provide an alternative explanation for the presence of calflagins in the flagellar blob after ablation of PFR2. It has been shown that PFR proteins such as PFR1 and the ROD1 antigen accumulate in the blob (Bastin et al., 1999b) and that numerous proteins with calcium binding ability are components of the PFR (Ridgley et al., 2000; Portman et al., 2009). A concentration of unregulated calcium binding proteins in the blob may therefore reduce the available calcium and cause calflagins to dissociate from the membrane and concentrate in that area. If there is an increased localisation of calflagins to the tip of the new flagellum (although to our knowledge this has not been demonstrated in procyclic form *T. brucei*) this effect may be exacerbated.

Calmodulin itself has also been shown to be a component of the PFR in a series of experiments by Ridgley et al. (2000). An antibody that recognised *T. brucei* calmodulin (Tryp-calmodulin) labelled the flagellum in both bloodstream and procyclic life-cycle stages with weaker signals associated with cytoplasmic vacuoles. Cryo-section immunogold showed that Tryp-calmodulin associates mainly with the PFR at the proximal and distal domains and on the filaments that connect the proximal domain to the FAZ. Gold particles were also observed along the axoneme consistent with localisation to the dynein arms and radial spokes. A possible interaction between Tryp-calmodulin and PFR1/2 was investigated by passing purified PFR proteins over a calmodulin-Sepharose column in the presence of calcium. Approximately 60% of the protein pool associated with the column with the remainder washing through. When the eluate from this first round was passed over a second column all of the protein was bound. This suggests that there are two pools of PFR proteins that are differentiated by their ability to bind calmodulin, although the presence in the preparations of proteins of similar molecular weight such as PAR1 and PAR4 homologues may also explain these results. PFR protein binding by calmodulin-Sepharose was sensitive to competition by the calmodulin binding peptide from calmodulin kinase II but not an irrelevant peptide, suggesting that there is a specific interaction between calmodulin and components of the PFR.

### 2.5 A PFR proteome

**Fig. 2:**
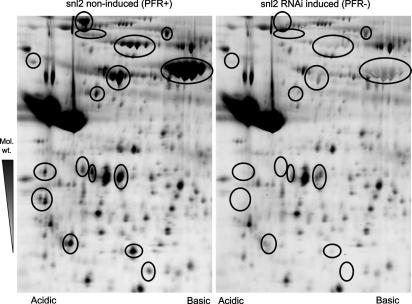
Two-dimensional gel comparison of purified *Trypanosoma brucei* cell line *snl2* flagella reveals at least 14 spots with reduced intensity after RNA interference (RNAi) induction. Flagella were purified using detergent and salt as in (Schneider et al., 1987). First dimension – isoelectric focussing over pH 3–pH 11 non-linear immobilised pH gradient strip (GE Healthcare). Second dimension – separation by 10% acrylamide SDS–PAGE. Samples were labelled with cy5 or cy3 and visualised on a Typhoon scanner (GE Healthcare).

Our increasing knowledge of the molecular complexity of the PFR suggests a role for this structure in a variety of functions within the flagellum. A fuller understanding of these roles will require an expansion of our knowledge of the protein components contributing to these functions. To this end we expanded on our earlier proteomic analysis (Pullen et al., 2004) of the *snl2* mutant cell line and used cutting edge comparative proteomics techniques (difference gel electrophoresis – DIGE (Fig. 2); and isobaric tags for relative and absolute quantitation – iTRAQ) to produce a PFR proteome consisting of 30 proteins (Portman et al., 2009). The proteins in this cohort include PFR1, PFR2, PAR1, PFR5, ADKA, ADKB, Tb5.20 and calmodulin which have all previously been proposed as PFR components. Interestingly, several other components discussed above, including some with strong evidence for a PFR localisation, were not identified in this study. There are many likely reasons for the absence of these components from the PFR2-dependent cohort: low abundance, presence in the inner sub-domain of the PFR that is still assembled in the absence of PFR2, or protein/peptide characteristics that effect detection in gel-based and/or mass spectrometric analyses. In addition to the previously identified components, 20 proteins in the PFR2-dependent cohort (paraflagellar rod proteome components – PFCs) were proteins of unknown function that have not previously been associated with the PFR. Amongst these were further examples of potential structural, regulatory and calcium interacting proteins as well as several for which a predicted function remains elusive. By following the logic loop and repeating our RNAi/comparative proteomic analysis with newly identified PFR components, we were able to show a number of dependency relationships between proteins within the PFR2-dependent cohort (Portman et al., 2009; Lacomble et al., 2009). One of these dependency networks involved the two novel PFR proteins PFC1 and PFC15 and the previously identified PFR adenylate kinases TbADKA and TbADKB. RNAi ablation of either PFC1 or PFC15 resulted in a loss of both of these proteins from purified flagellar fractions as well as decreases in the volume of spots corresponding to TbADKA and TbADKB. Most intriguingly, our bioinformatic analyses of PFC1 and PFC15 predicted that both contain Pfam domains associated with calcium signalling and sensing. PFC1 had a weakly predicted EF-Hand calcium binding domain and PFC15 was predicted to contain an IQ-calmodulin binding domain. This potential association of calcium signalling and adenine nucleotide homeostasis provides tantalising insights into the regulatory and functional networks operating within the PFR and implicates this structure in the wider regulation of flagellar function.

## 3 Functions of the PFR

The function of the PFR had been the subject of much conjecture. Early ideas tended to focus on concepts involving the PFR as a physical attribute to the flagellum i.e. as a thickening or stiffening agent (Fuge, 1969). The PFR has been implicated in the ubiquitous attachment of kinetoplastid parasites to the insect host at some stage in their life-cycle. Attachment is achieved via electron-dense hemi-desmosome-like plaques associated with the proximal portion of the flagellum which is enlarged and contains filaments that resemble PFR material and in some cases appear to emanate from the PFR structure (reviewed by Bastin et al., 1996b; Maga and LeBowitz, 1999). Further investigation of the function of the PFR in this process requires cell lines competent for establishing an infection in the insect host.

Real progress on function was initiated with two studies in *Leishmania* and *T. brucei* using reverse genetics to ablate the PFR and hence address mutant phenotypes (Santrich et al., 1997; Bastin et al., 1998). A PFR2 null mutant in *L. mexicana* and a PFR2 RNAi knockdown mutant in procyclic *T. brucei* (named *snl1*) produced markedly similar phenotypes. Despite some important differences between these organisms and mutants, some overall general observations emerged. Cells showed a dramatic decrease in flagellar wave frequency and amplitude that corresponded to a loss of forward cell motility. Bending patterns became unstable and wavelengths were shortened although the tip-to-base wave propagation pattern exhibited by trypanosomes (Walker, 1961) was preserved (Supplementary Movies S1 and S2). TEM of each mutant revealed a loss of the bulk of the PFR structure corresponding to the distal and intermediate domains and part of the proximal domain where it was not attached to the axoneme (Fig. 1D). Immunofluorescence staining consistent with PFR1 being present in the PFR remnant was also observed. This result predicted that of the two most abundant PFR components only PFR1 was required to form the extreme proximal domain and a subsequent study (Maga et al., 1999) confirmed this using a PFR1 null mutant of *L. mexicana*. In this cell line, none of the domains of the PFR assembled and PFR2 protein was transported to the flagellum but not incorporated. The fibres that attach the axoneme to the PFR still formed and their presence in both null mutant cell lines as well as a double null mutant of PFR1 and PFR2 shows that these proteins are not involved in these structures. The blob that accumulates during new flagellum formation in the procyclic *T. brucei* *snl1* mutant was shown to be an accumulation of PFR proteins that included PFR1. This suggests that in the absence of PFR2, PFR1 and other PFR components cannot be correctly assembled and accumulate at the flagellar tip. These studies confirmed that a correctly assembled PFR was necessary for normal flagellar function in these organisms. The availability of the mutant cell lines has enabled subsequent studies into the dynamics and regulation of flagellar assembly, the functions and components of the PFR and possible avenues of intervention against the pathologies of many trypanosomatid parasites.

It is important to note that both the original procyclic *T. brucei* *snl1* mutants and the inducible RNAi *snl2* mutant are viable (Bastin et al., 1998, 2000). However, Hunger-Glaser and Seebeck (1997) were unable to obtain a PFR2 double-knock-out cell line in procyclic *T. brucei*. This suggests a need for at least a low level of PFR2 expression in this organism that can still be achieved under RNAi conditions, although this protein was at levels undetectable by Western blot. Of course the failure to obtain knock-out of the second copy of a gene cannot prove its essential nature; however this experience may suggest an intrinsic difference in the essentiality of the PFR between procyclic *T. brucei* and *Leishmania*. In this respect the fact that *T. brucei* has a flagellum attached to its cell body along its length does offer an explanation for a difference. Until an inducible ectopic knock-out approach is used this must remain conjecture.

Trypanosomatids have an unusual tip-to-base flagellar beat initiation pattern (Walker, 1961) and Branche et al. (2006) describe an essentially normal wave amplitude at the distal tip of the procyclic form *T. brucei* flagellum that is not effectively propagated to the base after PFR2 ablation. These authors also observed rapid alterations between forward and reverse waves with the previously observed lack of cellular displacement. Finally, cell morphology was altered whereby the majority of cells no longer display the characteristic left-handed helical twist of the flagellum around the cell body but rather show a planar ‘L’ shape as a result of a near 90 degree bend in the cell body and attached flagellum. This work in procyclic form trypanosomes led these authors to propose a role for the PFR in generating the helical path of the flagellum around the cell body. In contrast, in the absence of a PFR, *T. brucei* bloodstream form cells still maintain this helical pattern despite becoming monstrous and otherwise contorted (Broadhead et al., 2006; Gadelha et al., 2007). This difference between the life-cycle stages alerts us to the importance of paying particular attention to the life-cycle stage being studied when assessing the form, extent and penetrance of RNAi mutants in *T. brucei*.

Following on from the previous point, a general observation can be made that flagellar mutations as a result of RNAi in procyclic forms may or may not result in cytokinesis and cell morphogenesis failures (Bastin et al., 1998; Hutchings et al., 2002; Dawe et al., 2005; Branche et al., 2006; Broadhead et al., 2006; Ralston et al., 2006; Baron et al., 2007; Farr and Gull, 2009) depending upon the protein under investigation. However, to our knowledge, all RNAi-knockdown mutations of flagellar proteins in the bloodstream form studied to date produce a lethal phenotype that manifests as aberrations in cytokinesis and abnormal cell morphology (Branche et al., 2006; Broadhead et al., 2006; Ralston and Hill, 2006; Farr and Gull, 2009). In the following section these distinctions are exemplified in the context of the PFR and are subsequently discussed as a more general phenomenon of flagellar proteins.

**Fig. 3:**
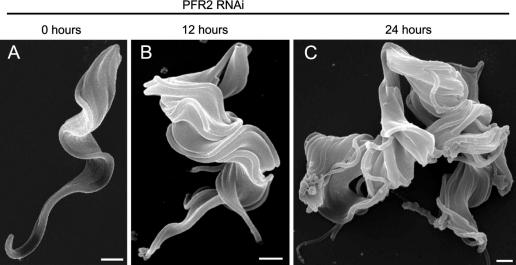
Scanning electron micrograph showing *Trypanosoma brucei* bloodstream form morphology (A) before, (B) 12 h after and (C) 24 h after RNA interference (RNAi) ablation of paraflagellar rod protein 2 (PFR2). Cells were processed as previously described (Broadhead et al., 2006). Bar = 1,000 nm.

Reproduced from Broadhead, R., Dawe, H.R., Farr, H., Griffiths, S., Hart, S.R., Portman, N., Shaw, M.K., Ginger, M.L., Gaskell, S.J., McKean, P.G., Gull, K., 2006. Flagellar motility is required for the viability of the bloodstream trypanosome. Nature 440, 224–227.

In the general terms of the PFR, our many studies using RNAi against PFR2 have shown that procyclic form cells exhibit a strong reduced-motility phenotype but are normal or near normal in their growth rate with no distinctive morphological defects other than the lack of a major portion of the PFR. However, recent work involving our group used doxycyclin-inducible RNAi against PFR2 in *T. brucei* to show that in contrast to the paralysed but viable procyclic form phenotype, ablation of PFR2 is lethal in the mammalian bloodstream form both in vitro in culture and in vivo in mouse infections (Broadhead et al., 2006; Griffiths et al., 2007) (Fig. 3). When the PFR is not correctly assembled, bloodstream form cells progress through multiple rounds of organellar replication but are unable to complete cytokinesis (Broadhead et al., 2006). This results in monstrous cells with multiple nuclei, kinetoplasts and flagella. Analysis at an early timepoint in the induction showed that motility is compromised before cytokinesis defects become apparent in the culture (Griffiths et al., 2007). Mice infected with an inducible PFR2 RNAi mutant developed parasitaemia as normal but when doxycyclin was added to the drinking water, thus inducing RNAi, infections were cleared within 16 h and did not re-occur during a subsequent 7 day monitoring period (Griffiths et al., 2007). There are many examples from a variety of experimental organisms where alterations in culture conditions, turbulence and other physical and biochemical factors produce different phenotypes for the same mutant (for examples see De Lozanne and Spudich, 1987; Larochelle et al., 1996; Brown et al., 2003; Ralston et al., 2006). The in vivo experiments described by Griffiths et al. (2007) were therefore vitally important to establish that the different environmental conditions experienced by the parasite in the bloodstream, either in terms of turbulence in blood vessels or composition of growth medium, could not rescue the lethal phenotype observed in culture. The success of this study validates flagellar function as an important new target for possible intervention against *T. brucei* infections.

We also showed (Broadhead et al., 2006) that RNAi mutants affecting four different axonemal components exhibit the differential phenotype between the bloodstream and procyclic forms that we observed after PFR2 ablation. This demonstration that individual ablation of different proteins in different flagellar compartments led to the same growth effect, cell morphogenesis defects and defined terminal phenotype argued for a general and critical requirement for normal flagellar function in the bloodstream form. Later in the same year work from other groups revealed a requirement for another axonemal protein, Trypanin (Ralston and Hill, 2006), in the bloodstream but not procyclic form, and again demonstrated the strikingly different effects of PFR2 RNAi between the two life-cycle stages (Branche et al., 2006). Recently our ablation of the TAX-2 protein in the two life-cycle stages added another axonemal protein to this growing list presenting the differential phenotype (Farr and Gull, 2009). The range of components for which this differential requirement has been observed adds weight to the view that loss of normal flagellar function, and not a specific requirement for the PFR, is the major factor in the lethality of PFR2 ablation in the bloodstream form. It is important to note here that impairment of cytokinesis in the bloodstream form can be achieved by RNAi ablation of a wide range of flagellar proteins with a range of effects on the structures and functions of the flagellum. This reliance on correct flagellar function in the bloodstream form may reflect a dependency on either natural beat, the translation of natural beat to coordination of separation of a late dividing cell or possibly the dependence of an internal cytoskeletal structure on a physical flagellar component rather than progressive cell movement. Analysis of these phenotypes requires a reasonably sophisticated level of knowledge of the role of the ablated protein component in the parasite cytoskeleton. Again, the debate here has to recognise that explanations for a general collective category of ‘bloodstream lethal but procyclic viable’ may vary and these are discussed later. The restricted evolutionary distribution of the PFR structure and components compared to the more conserved structure and components of the axoneme makes the PFR a particularly valuable target possibility for therapeutic intervention.

### 3.1 The role of flagellar motility in *T. brucei* cytokinesis

Bloodstream form *T. brucei* appear to be exquisitely sensitive to ablation of flagellar components and appear to rely on flagellar function to complete cytokinesis. A number of recent studies have demonstrated the contrast between phenotypes associated with ablation of individual flagellar components in procyclic and bloodstream form *T. brucei* (Branche et al., 2006; Broadhead et al., 2006; Ralston and Hill, 2006; Ralston et al., 2006; Farr and Gull, 2009). All of these groups are in agreement that flagellar function in procyclic form cells can be disrupted by ablation of individual flagellar components with little or no impact on the growth rate of the cell population. However, when the same proteins are ablated in the bloodstream form, cells become monstrous and contorted with multiple nuclei, kinetoplasts and flagella indicating a catastrophic failure in cytokinesis. The underlying molecular mechanisms behind this dichotomy remain unclear although numerous differences between bloodstream form and procyclic form cells could be implicated and indeed, this may be a combinatorial or pleiotropic phenomenon. Our laboratory has recently completed a full description of the normal mechanisms of division and cytokinesis in bloodstream and procyclic form cells (Vaughan and Gull, unpublished data). There are many differences between these cell types: organelle segregation patterns; cleavage point initiation; the presence of the canonical flagella connector in procyclic but not bloodstream forms; that all point to contrasting patterns that could be differentially influenced by flagellar mutants. Also under culture conditions the bloodstream form cell cycle is significantly faster than that of commonly used laboratory strains of procyclic form and it may be that this time difference allows these procyclic form cells greater latitude in the final stages of cell division. An increase in the time taken to complete cytokinesis could therefore have lethal consequences for the proper segregation and positioning of organelles and cleavage furrows in the bloodstream form but not the procyclic form.

Bloodstream form cytokinesis failure has been demonstrated in RNAi mutants with a diverse range of effects on the flagellum. For example, in the procyclic form, ablation of PFR2 results in the loss of most of the PFR structure (Bastin et al., 1998) whilst ablation of the flagellar protein TbPACRGA has very little effect on the morphology of the flagellum due to redundancy with TbPACRGB (Dawe et al., 2005). However, despite the differences in procyclic form phenotype, ablation of either PFR2 or TbPACRGA alone is lethal to the bloodstream form trypanosome (Broadhead et al., 2006). Ablation of Tbmbo2, DIGIT and Trypanin result in viable cells with qualitatively different motility defects in the procyclic form (Hutchings et al., 2002; Broadhead et al., 2006) and yet produce equivalent lethal phenotypes in the bloodstream form (Broadhead et al., 2006; Ralston and Hill, 2006). Finally, a homologue to the trypanosome axonemal protein TAX-1 (Broadhead et al., 2006) was shown to be a small component of one of the various inner arm dynein complexes in *Chlamydomonas* (Yamamoto et al., 2006). Ablation of this single component in procyclic form *T. brucei* does not interfere with overall axoneme structure (Farr, H.K., 2007, DPhil Thesis, The Eukaryotic Flagellum in Health and Disease, University of Oxford, UK) and does not compromise cell growth (Broadhead et al., 2006) yet produces the dramatic lethal phenotype on ablation in bloodstream form trypanosomes (Broadhead et al., 2006).

Productive movement of the cell has been shown to be an essential mechanism in the clearance of the variant surface glycoprotein (VSG) from the cell surface into the flagellar pocket in bloodstream form trypanosomes (Engstler et al., 2007). VSG is the effective molecule in trypanosome antigenic variation, a strategy for avoiding the host immune system, and is one of the most abundant proteins in the bloodstream form cell (for reviews on VSG see Pays et al., 2004; Taylor and Rudenko, 2006). It is possible that if the process of VSG clearance is defective, endo- and exocytotic processes internalising VSG and delivering newly synthesised protein to the cell surface will be impaired, leading to the vastly enlarged flagellar pocket morphology observed in flagellum mutants. These enlarged pockets would provide unassailable physical challenges to the precise morphogenetic patterning required for trypanosome cytokinesis.

There is evidence that aberrant flagellar structure and function can also impair proliferation and morphogenesis in procyclic forms. Simultaneous ablation of the two homologues of PACRG in procyclic form *T. brucei* results in reduced growth rates compared to wild-type populations (Dawe et al., 2005). These double-knock-down cells exhibit defects in kinetoplast positioning, paralysed flagella and disruptions to the outer doublet microtubular ring of the axoneme. The contribution of each of these defects to the reduction in growth rate is not currently known but a reduction in cell motility is an early manifestation in this phenotype. Some studies have demonstrated the aggregation of *T. brucei* cells after RNAi ablation of flagellar proteins under certain culture conditions (Branche et al., 2006; Ralston et al., 2006; Baron et al., 2007). In these cases, the slow-down in growth observed could be rescued by agitating the cultures, suggesting that cell motility, either as a direct effect or as a secondary effect (e.g. cells becoming locally concentrated due to settling in the culture flask), plays a role in the growth rate of cell populations. However, there are discrepancies in the procyclic form growth phenotypes reported in the literature (Branche et al., 2006; Broadhead et al., 2006; Ralston et al., 2006; Baron et al., 2007). Use of different growth media in different laboratories and treatment of the culture are likely variables that would need to be controlled in order to conduct an accurate comparison. As trypanosome cell density follows a classical microbiological growth curve in culture with lag, log and stationary phases, it is important to analyse any studies of population growth with these phases in mind. To our knowledge, rescue of bloodstream form motility mutants by agitation of the culture medium has not yet been demonstrated. However, work from our group has shown that turbulence in the mammalian host bloodstream is not sufficient to rescue the phenotype (Griffiths et al., 2007). It is possible that it is the specific auger-like twisting motility of *T. brucei* that is necessary for the completion of cytokinesis and that turbulence in the mammalian blood stream does not sufficiently provide this.

As well as motility functions, most if not all flagella have sensory/regulatory functions. These translate to an influence for flagella in many cellular processes (Ginger et al., 2008). There is evidence that cilia and flagella act as part of specific cell cycle check points in a number of organisms with *C. reinhardtii* being the best studied. Recently the role of the flagellum as an instrument of signal transduction, including an involvement in Hedgehog signalling, has excited much interest (for reviews see Scholey and Anderson, 2006; Pedersen and Rosenbaum, 2008). It may be that loss of flagellar motility in bloodstream form *T. brucei* interferes with critical signalling pathways involved in cell cycle progression. It should be noted that in all of the motility mutants that have so far been shown to cause lethality in the bloodstream form, the flagellum is still built, raising the possibility of some requirement for correct flagellar function in a flagellar-derived signalling pathway. The role of cilia and flagella in cell cycle regulation has also been discussed from the point of view of the sequestration (as the flagellar basal body) of one of the centrioles needed for mitotic spindle formation (Pan and Snell, 2007; Ralston et al., 2009). The trypanosome flagellum, however, unlike that of *Chlamydomonas* or the vertebrate primary cilium, is not resorbed during the cell cycle (Sherwin and Gull, 1989) and the basal body is not required for mitotic spindle formation, so even if this specific biology of a flagellum or cilium exists in other cells it is unlikely to be a factor in the cytokinesis defects observed in bloodstream form motility mutants.

### 3.2 Speculations on the role of the PFR in motility

The mechanisms underlying the role of the PFR in flagellar motility are becoming clearer as the result of recent component analyses. Ablation of the PFR in both *T. brucei* and *L. mexicana* demonstrated that this structure is essential for maintaining flagellar motility (Santrich et al., 1997; Bastin et al., 1998). Early proposals suggested that the PFR contributes to the flagellar beat by increasing stiffness (Fuge, 1969). It is possible that recently identified signalling and metabolic enzymes built into the PFR lattice could induce conformational changes in the structure as a response to stimuli making it permissive for different forms of flagellar beat. As a possible counterpoint to this argument it has recently been shown that the flagellum of *C. deanei*, which possesses a reduced PFR alongside the proximal third of the axoneme, produces waveforms with comparable curvature and wavelength to that of *C. fasciculata* which possesses a PFR along the length of the flagellum (Gadelha et al., 2007). *Crithidia deanei* is capable of producing both ciliary and flagellar beat patterns and there is no observable difference between the curvature of the beat at the proximal and distal ends of the flagellum (Gadelha et al., 2007). Another possible role for the PFR in flagellar motility is as a phosphotransfer relay to maintain the supply of ATP to the more distal portions of the flagellum more effectively than by diffusion from the cell body (for reviews see Oberholzer et al., 2007a; Ginger et al., 2008). The discovery of two PFR-specific adenylate kinases (Pullen et al., 2004) supports this hypothesis. The discovery of other enzymatic and Ca^2+^ regulated components and the possible link between these and adenine homeostasis (Bastin et al., 1996b; Maga and LeBowitz, 1999; Ridgley et al., 2000; D’Angelo et al., 2002; Ginger et al., 2005; Oberholzer et al., 2007b; Portman et al., 2009) now points to a role for the PFR as a regulatory and metabolic platform for control of both motile and sensory flagellar functions. We suggest that a cyclic nucleotide signalling pathway, alongside components necessary for adenine nucleotide homeostasis, is hardwired into the structure of the PFR and is regulated spatially and temporally, perhaps in part by Ca^2+^, to avoid cross-talk between overlapping substrate sets. The specificity and effectiveness of signalling pathways is often reliant on the anchoring of effecter proteins into complexes or structures (reviewed by Pawson and Scott, 1997) that can provide localised domains of messenger molecules and appropriately limit the signal cascade. Within the confined domain of the eukaryotic flagellum, the complex adenine nucleotide metabolism employed to fulfil regulatory, signalling and energy requirements suggests the need for a high level of order. For example, there are well established roles for calcium signalling and adenine nucleotide homeostasis in the regulation of dynein arm function and flagellar beat pattern (Bonini and Nelson, 1988; Bonini et al., 1991; Yagi, 2000; Inoue and Shingyoji, 2007). The presence of proteins involved in calcium signalling and adenine nucleotide homeostasis could therefore implicate the PFR in the calcium-regulated control of flagellar waveform, a phenomenon reported in numerous organisms including trypanosomes.

One might predict that ablation of critical proteins within a potential PFR regulatory network would result in changes in the ability of the flagellum to propagate particular waveforms. Our initial unpublished observations of cells after ablation of PFC15 have so far not shown gross changes in motility and previous studies of RNAi ablation of ADKA and ADKB in *T. brucei* did not detect any effects on the motility of the cells (Pullen et al., 2004). It is likely that the techniques used to date to analyse motility have not been sufficiently sensitive to detect subtle changes in the motility of RNAi-induced cells. For example, most studies analyse progressive cell movement or sedimentation of cells rather than beat characteristics. It is conceivable that the ‘default setting’ for the PFR is to normal tip-to-base flagellar beating and that ablation of critical proteins in the PFR regulatory network causes an inability of the cell to produce rare waveforms such as ciliary beating and wave reversal (Gadelha et al., 2007). It is also possible that, given the apparent requirement for motility in certain life-cycle stages, redundant systems are in place or that any changes in flagellar motility induced by ablation of PFR proteins are not observable in cultured cells. These questions remain to be answered and we are currently developing techniques to more fully explore the role of the PFR in motility.

We propose that in addition to the homeostatic and metabolic roles, the PFR acts as a site for integrating and transmitting external signals detected by the flagellum (this organelle has a well established role in environment sensing and cell signalling in a variety of organisms) to the axoneme or cell body. The earlier discussion of the presence of proteins involved in cAMP production and regulation both on the flagellar membrane and as hardwired components of the PFR provides clues to the pathways involved.

## 4 Regulation of the PFR in the cell cycle and life cycle

Kinetoplastid parasites have a complex interaction of life-cycle and cell cycle, often involving both host and vector interactions. The PFR is a feature of all life-cycle stages with the exception of the amastigote form of *T. cruzi* and *Leishmania* spp. where the reduced flagellum does not emerge from the flagellar pocket and does not present a PFR. The flagellum of Kinetoplastida is built over only a proportion of the cell cycle. Thus both life-cycle and cell cycle considerations raise issues as to how regulation of gene expression is achieved. Are all flagellar proteins made even when not used or are there discrete life and cell cycle regulatory processes?

### 4.1 Cell cycle regulation

A new flagellum is built once during the trypanosomatid cell cycle while the existing (old) flagellum persists. The PFR begins to form after 0.52 of the cell cycle in *T. brucei* (Sherwin and Gull, 1989). The dynamics of PFR formation and some of the factors involved have been investigated using the *snl1* constitutive PFR2 RNAi mutant (Bastin et al., 1999b) and an inducible Ty epitope tagged PFR2 cell line (Bastin et al., 1999a). As previously described, a striking feature of the *snl1* phenotype was the blob (Bastin et al., 1999b). Careful observation of the blob (Bastin et al., 1999b) revealed that it increased in size as the new flagellum extended, suggesting the involvement of an intraflagellar transport (IFT, Kozminski et al., 1993) anterograde transport mechanism. At some point after cytokinesis, but before the formation of a new flagellum, the blob was removed (the new flagellum becoming the old flagellum in subsequent rounds of the cell cycle). Removal of the aggregate implicated the retrograde IFT process and a level of cell cycle regulation. An additional level of control may involve cell cycle control exercised over the synthesis of new flagellar components. Another interesting observation was that although PFR1 protein was still transported to the distal site of incorporation, other PFR components were not. The ROD1 antigen was almost completely absent from the new flagellum and rather remained in the cytoplasm at levels much reduced compared to wild-type. Stably transfected cell lines expressing an inducible Ty epitope tagged PFR2 allowed the pattern of tagged protein incorporation to be assessed (Bastin et al., 1999a). The results point to a major site of new protein incorporation at the distal tip of the new flagellum and a minor site of incorporation along its length, as well as incorporation of new protein into the old flagellum that is consistent with a low level of protein turnover after the PFR has been built.

A question arises as to whether there are specific targeting or retention signals within the PFR or flagellar protein sequences. Tagged PFR2 deletion mutants indicate that a region of the PFR2 protein between amino acids 514 and 570 is necessary for flagellar localisation but is not sufficient to exclusively target GFP to the flagellum (Bastin et al., 1999a). A further set of deletion mutants defined a region of seven amino-acids between residues 564 and 571 that is required for incorporation at the distal tip of the new flagellum but not along the length. In subsequent work, this region was further refined to the tri-peptide HLA and was shown to also be necessary for targeting the axonemal component Tryp-ARP to the flagellum (Ersfeld and Gull, 2001). The N-terminal sequences of the *T. brucei* adenylate kinases discussed earlier (Pullen et al., 2004) have extended our knowledge of signals critical to flagellar localisation.

### 4.2 Life-cycle regulation

Gene regulation in Kinetoplastida is predominantly achieved post-transcriptionally and primary RNA transcripts are, in the main part, likely transcribed uniformly across the genome in a poly-cistronic manner (discussed by Clayton and Shapira, 2007). The amastigote life-cycle stages of *T. cruzi* and *Leishmania* spp. lack a PFR and several studies have shown that PFR1 and PFR2 mRNA levels are enriched between 10- and 12-fold in *L. mexicana* promastigotes compared to amastigotes (Mishra et al., 2003; Holzer et al., 2006, 2008). In the first of these studies it was shown that transcription rates across the PFR2 locus (that contains three tandem copies of the PFR2 open reading frame in this organism) were nearly constant in both life-cycle stages supporting the unregulated production of a polycistronic precursor RNA. The half-life of the mature transcripts differed significantly between life-cycle stages, being 3.9 h in promastigotes and 0.8 h in amastigotes, suggesting that mRNA stability is an important regulatory mechanism. Analysis of mutant cell lines facilitated the definition of a 10 nucleotide element that was required for regulation of transcript level and was sufficient to confer regulation on a transcript with a vector-derived exogenous 3′ untranslated region (UTR). This element was named the Paraflagellar Regulatory Element (PRE). This work continued with the screening of the *L. major* genome for instances of the PRE (Holzer et al., 2008). A total of 343 occurrences in the genome were identified, 139 of which fell on the coding strand within 2,000 bp of an open reading frame stop codon. Of these, 78 were within 500 bp of a stop codon and are therefore very likely to be contained within the 3′ UTR of the associated transcript. Not all of the genes associated with the PRE have a predicted function that is immediately consistent with the PFR although proteins involved in flagellar organisation and calcium or calmodulin binding were identified. Five of these proteins were selected for further study and all showed down-regulation in the amastigote form. The PRE is found in orthologous proteins from other *Leishmania* spp., which, together with the fact that not all of the open reading frames identified encode PFR proteins, points to a conserved mechanism for down-regulating a variety of mRNA transcripts in the amastigote form.

Further insights into the regulation of PFR proteins in the different life-cycle stages of *Leishmania* spp. have been provided in a series of recent reports from Wiese et al. (Wiese et al., 2003a,b; Erdmann et al., 2006). The mitogen-activated protein kinase kinase, LmxMKK, was found to be down-regulated in the amastigote form of *L. mexicana*. LmxMKK null mutants showed a dramatic phenotype whereby cell motility was severely compromised and flagellar length was reduced by at least 80% with a majority lacking a PFR. These cells were able to form lesions after injection into mice and the resulting amastigotes presented the typical short flagellum. Null mutants of the kinase LmxMPK3, which has a similar expression profile to LmxMKK, also result in short flagella with either an absent or reduced PFR (Erdmann et al., 2006). Interestingly no phosphorylated LmxMPK3 could be found in the LmxMKK null mutant, suggesting that these kinases operate in the same signalling pathway and that LmxMPK3 is a substrate of LmxMKK. One of the main differences between the phenotypes of the null mutants was the presence of PFR2 protein. In the LmxMKK null mutant no PFR2 protein could be detected whereas in LmxMPK3 null variable amounts of PFR2 could be detected in the flagellum although evidently this was not assembled into a PFR. This raises an interesting question of whether LmxMKK has additional substrates, perhaps involved in the expression of proteins such as PFR2. We suggest that this could possibly be achieved through the inactivation in promastigotes of a factor responsible for degrading mRNA that carries the PRE discussed earlier.

Recent work from Tammana et al. (2008) proposes a role for actin in building the flagellum and particularly the PFR in *L. donovani*. Mutant cells carrying either a double or single knock-out of the *L. donovani* actin-depolymerising factor, LdCof, exhibit a similar phenotype to that observed for null mutants of LmxMKK and LmxMPK3. These authors propose that LdCof/actin dynamics are involved in the transport of flagellar components to the base of the flagellum prior to intraflagellar transport, hence the shortened flagellum phenotype observed in LdCof knock-out mutants, and that there is a specific role for LdCof in the formation of the PFR as this structure is completely absent from null mutant flagella. Based on the similarity of the LdCof null phenotype to those reported for LmxMKK and LmxMK3, these authors go on to hypothesise that in the absence of LIM kinase (an enzyme not coded in the *Leishmania* genome which is responsible for the reversible activation of ADF/cofilin in other eukaryotic systems) LdCof is a substrate of the Mitogen-activated protein kinase pathway that includes homologues of LmxMKK and LmxMPK3.

## 5 Immunogenicity of PFR proteins

Infection by trypanosomatid parasites is endemic in many of the poorest areas of the world and existing treatments are associated with high toxicity and increasing drug resistance, leading to an urgent need for modern therapies (de Souza, 2007; Checchi and Barrett, 2008; Simarro et al., 2008 for current perspectives). There are currently no vaccines available although work from several groups over the last decade or so has identified PFR proteins as attractive targets for generating protective immunity against several species of trypanosome (Wrightsman et al., 1995, 2002; Miller et al., 1996, 1997; Wrightsman and Manning, 2000; Luhrs et al., 2003; Michailowsky et al., 2003; Saravia et al., 2005). Immunisation with PFR proteins purified from *T. cruzi* epimastigotes was shown to completely protect mice from a subsequent challenge by an otherwise fatal dose of this parasite (Wrightsman et al., 1995). The protection conferred was dependent upon the delivery method of the PFR vaccine. Intraperitoneal injections completely failed to protect immunised mice while s.c. injections conferred immunity. Chagasic serum from human patients was also shown to contain antibodies that recognised PFR proteins (Wrightsman et al., 1995; Michailowsky et al., 2003). In a subsequent report (Wrightsman and Manning, 2000) it was shown that PFR antigen co-adsorbed onto alum with recombinant IL-12 or adenovirus-expressed IL-12 could elicit a Th1-type cell-mediated immune response that provide protection against subsequent infection when delivered subcutaneously. The use of recombinant PFR proteins as antigens confirmed that it was these and not some previously unidentified contaminant of the preparations that conferred immunity (Luhrs et al., 2003). Other recombinant PFR and DNA vaccine approaches have led to a deeper understanding of the cellular mechanisms of immunity to *T. cruzi* infection (Wrightsman et al., 2002; Michailowsky et al., 2003; Morell et al., 2006).

PFR2 has also been shown to have potential as a vaccine against *Leishmania* infections (Saravia et al., 2005). A *pfr-2* DNA vaccination led to delayed lesion appearance or significant lesion reduction in male hamsters after subsequent challenge with 10,000 and 500 *L. mexicana* promastigotes. Interestingly this same treatment exacerbated subsequent lesions when applied to female hamsters. Immunisation with recombinant PFR2 protein prevented *Leishmania panamensis* lesion formation in female hamsters but was ineffective against *L. mexicana*. However, a prime boost immunisation regime using both DNA and recombinant protein reduced lesion size in female hamsters challenged with 500 *L. mexicana* promastigotes.

An interesting question arises as to how an intracellular antigen that is associated with a structure apparently not present in the mammalian form of the parasite can elicit an immune response. Serum from infected humans or animals can recognise PFR proteins and a mechanism has been proposed whereby the degradation of the flagellum and PFR during the promastigote to amastigote transition makes components available for recognition by the immune system (Michailowsky et al., 2003; Saravia et al., 2005). Another possibility is that lysis of a portion of the parasite cells could expose internal antigens to the host. This awaits experimental verification.

## 6 Wider perspectives on the PFR

Although the PFR is a unique structure restricted to Kinetoplastida and Euglenida, extra-axonemal structures in general are rather common. These structures can be symmetrically arranged around the axoneme or asymmetrically located as in the case of the PFR. In addition they can run along the full length or only a portion of the axoneme. They occur in many diverse organisms from mammalian and insect sperm to a range of protists such as dinoflagellates and *Giardia* (Talke and Preisfeld, 2002; Elmendorf et al., 2003; Werner and Simmons, 2008). Perhaps the most intriguing extra-axonemal structure to compare with the kinetoplastid PFR is the mammalian sperm Outer Dense Fibres and fibrous sheath. Several components of the flagellar accessory structures of mouse sperm have been identified. These include adenylate kinase and proteins involved in cAMP signalling drawing intriguing parallels to the PFR (reviewed by Oberholzer et al., 2007a). Thus the concept of the PFR as a metabolic, homeostatic, regulatory and sensory platform may well turn out to be a conserved phenomenon among extra-axonemal structures in evolution.

## Figures and Tables

**Table 1:** Orthologues of all identified paraflagellar rod (PFR) proteins in six species of kinetoplastid. Orthologues were determined by reciprocal BLASTP analysis to databases obtained from the relevant genome projects. Results were processed using custom Perl scripts and Microsoft Excel spreadsheets. Where multiple identical sequences are present in a genome, a single representative accession number has been provided.

Name	*Trypanosoma brucei*	*Trypanosoma congolense*^a^	*Trypanosoma cruzi*	*Leishmania major*	*Leishmania braziliensis*	*Leishmania infantum*
PFR1	Tb927.3.4290	1193c01.q1k_17	XP_809830	LmjF29.1750	LbrM31_V2.0160	LinJ29_V3.1890
PFR2	Tb927.8.4970	1087c12.p1k_0	XP_814169	LmjF16.1430	LbrM16_V2.1480	LinJ16_V31510
PAR1	Tb11.01.5100	Not found	XP_818742	LmjF09.1320	LbrM09_V2.1380	LinJ09_V3.1390
PAR4	Tb927.5.4480	865g03.q1k_12	XP_805825	LmjF05.0040	LbrM05_V2.0040	LinJ05_V3.0040
PFR5	Tb927.2.4330	999f08.p1k_5	XP_816074	LmjF27.1850	LbrM27_V2.1990	LinJ27_V3.1750
PFR6	Tb927.7.6970	1341e03.p1k_4	XP_819921	LmjF05.0920	LbrM05_V2.0900	LinJ05_V3.0920
Calmodulin	Tb11.01.4621	528h03.p1k_2	XP_808089	LmjF09.0910	LbrM09_V2.0960	LinJ09_V3.0980
ADKA	Tb927.2.5660	1144b06.p1k_5	Not found	Not found	Not found	Not found
ADKB	Tb10.70.7330	1389a06.q1k_3	XP_810144	LmjF21.1250	LbrM21_V2.1450	LinJ21_V3.1490
Gamma tubulin	Tb927.3.910	526h04.q1k_1	XP_814966	LmjF25.0960	LbrM25_V2.0840	LinJ25_V3.0990
KMP-11	Tb09.211.4512	Not found	XP_810488	LmjF35.2210	LbrM34_V2.2150	LinJ35_V3.2250
Myosin XXI	Tb11.01.7990	Not found	XP_809654	LmjF32.3870	LbrM32_V2.4110	LinJ32_V3.4020
PDEB1	Tb09.160.3590	857h01.q1ka_11	XP_820270	Not found	LbrM15_V2.1250	Not found
PDEB2	Tb09.160.3630	Not found	XP_804464	LmjF15.1480	LbrM15_V2.1480	LinJ15_V3.1550
Tb5.20	Tb11.01.6740	1557c01.q1k_0	XP_817636	LmjF32.1910	LbrM32_V2.2100	Not found
TbI17	Tb10.26.0960	1023a12.q1k_9	XP_811780	Not found	Not found	Not found
TbI2	Tb927.3.5310	1411c01.q1k_2	XP_811482	LmjF29.0350	LbrM29_V2.0290	LinJ29_V3.0360
PI3K-related kinase	Tb11.01.6300	Not found	XP_812942	LmjF32.1460	LbrM32_V2.1620	LinJ32_V3.1520
PFC1	Tb927.8.6660	1023a07.q1k_7	XP_806248	LmjF24.1560	LbrM24_V2.1300	LinJ24_V3.1630
PFC2	Tb927.8.3790	1148b02.q1k_15	XP_802628	LmjF10.0180	LbrM10_V2.0180	LinJ10_V3.0160
PFC3	Tb927.8.1550	1291d12.q1k_4	XP_816963	LmjF07.0310	LbrM07_V2.0320	LinJ07_V3.0470
PFC4	Tb927.6.4140	1085c12.p1k_4	XP_809400	LmjF30.2850	LbrM30_V2.2830	LinJ30_V3.2870
PFC5	Tb927.7.1920	1126c02.q1k_2	Not found	Not found	Not found	Not found
PFC6	Tb927.3.3770	1124g03.p1k_0	XP_808170	LmjF29.1170	Not found	LinJ29_V3.1260
PFC7	Tb927.3.3750	1407h03.q1k_4	XP_813907	Not found	Not found	Not found
PFC8	Tb927.6.3670	1335c05.q1k_7	XP_819095	LmjF30.2390	LbrM30_V2.2340	LinJ30_V3.2400
PFC9	Tb11.01.6510	Not found	XP_804149	LmjF32.1680	LbrM32_V2.1850	LinJ32_V3.1760
PFC10	Tb927.2.3660	1130f02.p1k_3	XP_812725	LmjF33.2780	LbrM33_V2.3060	LinJ33_V3.2920
PFC11	Tb927.2.2160	171e08.q1k_13	XP_807404	LmjF02.0310	LbrM02_V2.0360	LinJ02_V3.0280
PFC12	Tb11.02.2350	1278f02.q1k_13	XP_817448	Not found	Not found	Not found
PFC13	Tb927.2.950	771g11.q1k_1	Not found	Not found	Not found	Not found
PFC14	Tb10.6k15.0810	1004e12.q1k_6	XP_812195	LmjF36.4230	LbrM35_V2.4480	LinJ36_V3.4440
PFC15	Tb10.61.1260	Not found	XP_817255	LmjF19.0520	LbrM19_V2.0840	LinJ19_V3.0520
PFC16	Tb10.26.0680	1069e05.p1k_0	XP_819848	LmjF33.0610	LbrM33_V2.0650	LinJ33_V3.0660
PFC17	Tb11.01.3000	Not found	XP_803930	Not found	Not found	Not found
PFC18	Tb10.6k15.1510	410c11.p1k_1	XP_815947	LmjF36.5870	LbrM35_V2.6170	LinJ36_V3.6130
PFC19	Tb10.6k15.0140	758b01.p1k_15	XP_817130	LmjF36.4780	LbrM35_V2.5030	LinJ36_V3.5010
PFC20	Tb10.389.0100	1205b11.p1k_21	XP_819266	Not found	Not found	Not found
TczAC/GRESAG 4	Tb10.61.0280	189g01.p1k_3	XP_806389	LmjF17.0190	LbrM17_V2.0130	LinJ17_V3.0130
FCaBP	Tb927.8.5440	20a04.p1k_4	XP_808305	Not found	LbrM16_V2.0930	LinJ16_V30930
LdCof	Tb927.3.5180	1379b03.q1k_1	XP_810628	LmjF29.0510	LbrM29_V2.0450	LinJ29_V3.0520
LmxMKK	Tb927.3.4860	1242a01.q1k_13	XP_822045	LmjF29.2320	LbrM29_V2.2290	LinJ29_V3.2430
LmxMPK3	Tb927.8.3550	1097a03.q1k_20	XP_807815	LmjF10.0490	LbrM10_V2.0620	LinJ10_V3.0540

a When interpreting ‘Not found’ statements it is important to take into account the completeness of the relevant genome project.

## Appendix A Supplementary data

Supplementary Movie S1 Procyclic form *Trypanosoma brucei* cell line *snl2* in the absence of RNA interference induction. Cells are shown swimming in a culture flask in SDM79 medium supplemented with 10% FBS and 7.5 mg ml^-1^ Haemin.

Supplementary Movie S2 Procyclic form *Trypanosoma brucei* cell line *snl2* 48 h after induction of RNA interference by the addition of 1 μg ml^-1^ doxycyclin to the culture medium. Cells are shown swimming in a culture flask in SDM79 medium supplemented with 10% FBS and 7.5 mg ml^-1^ Haemin.
